# Intersectional inequalities in somatic symptom severity in the adult population in Germany found within the SOMA.SOC study

**DOI:** 10.1038/s41598-024-54042-8

**Published:** 2024-02-15

**Authors:** Rieke Barbek, Anne Toussaint, Bernd Löwe, Olaf von dem Knesebeck

**Affiliations:** 1 Institute of Medical Sociology, University Medical Center Hamburg-Eppendorf, 20246 Hamburg, Germany; 2 Department of Psychosomatic Medicine and Psychotherapy, University Medical Center Hamburg-Eppendorf, 20246 Hamburg, Germany

**Keywords:** Gender, History of migration, Income, Intersectionality, Social inequalities in health, SOMA.SOC, Somatic symptoms, Somatic symptom severity, Public health, Risk factors

## Abstract

Somatic symptoms are common in a wide range of medical conditions. In severe cases, they are associated with high individual and economic burden. To explore social inequalities in somatic symptom severity (SSS) and to identify social groups with highest SSS, we applied an intersectional research approach. Analyses are based on cross-sectional data of the adult population living in Germany (N = 2413). SSS was assessed with the Somatic Symptom Scale-8. A multiple linear regression model with three-way interaction of gender, income and history of migration and post-hoc pairwise comparison of estimated marginal means was conducted. Analyses revealed intersectional inequalities in SSS along the axis of gender, income, and history of migration. Highest SSS was found in males with low income whose parent(s) immigrated, females with low income who immigrated themselves, and females with low income and no history of migration. Intersectional approaches contribute to a more comprehensive understanding of health disparities. To reduce disparities in SSS, proportionate universal interventions combining universal screening and targeted treatment seem promising.

## Introduction

Somatic symptoms are one of the primary causes of medical consultations^1^. They appear in a wide range of physical, mental, and psychosomatic diseases like cancer^2^, coronary heart disease^3^, anxiety, depression^4,5^ or somatic symptom disorder^6^. Overall, the most frequent symptoms are exhaustion- and pain-related complaints^7,8^. Thereby, the vast majority of all somatic symptoms presented in primary care turn out to be either minor and self-limited (about 75%) or persistent (about 20%), whereas less than 5% are acutely serious^1^. Women, older aged people, and people with low socio-economic status more often report severe somatic symptoms^7,9–11^. Regarding race/ethnicity (or adapted to the German research context, history of migration) as another important social determinant of health, evidence is lacking. Severe somatic symptoms are associated with functional impairment, a lower quality of life, sick leave, and increased health care visits^5,8,9,11,12^. Adjusted for chronic illness, they are an independent predictor of ill health and mortality (in men)^9,10,13^.

To identify individuals with high somatic symptom severity (SSS), brief universal self-reported screening tools like the Somatic Symptom Severity Scale-8 (SSS-8) as the short form of the Patient Health Questionnaire-15 (PHQ-15) seem promising^9,14^. For targeted interventions, depending on the type and extent of somatic symptoms (self-limited or persistent), medication and exercises to reduce pain, fatigue or digestive problems^1,15^ as well as antidepressants and cognitive behavioral interventions turned out to be effective^1,16–18^. The interventional approach of combining universal screening and targeted treatment follows the proportionate universalism formulated by Marmot & Bell^19^ which seems most promising to reduce health disparities.

However, it is still unclear which social subgroups have highest SSS and therefore may need particular attention in proportionate interventions. As described above, research on SSS focuses on separate socio-demographic variables so far. However, to gain a comprehensive understanding of the social context of SSS, the interplay of relevant social determinants of health in SSS needs to be examined. According to the framework of social determinants of health developed by the World Health Organization^20^, income, education, gender, and race/ethnicity have proven to be key social predictors of poor health outcomes. Understanding the interplay of these (and further) social predictors is the objective of intersectional research^21^. Intersectionality can be understood as the interaction of multiple social categories leading to more than additive disadvantage, discrimination and inequality. The framework is based on the work of Kimberlé Crenshaw who initially criticized the double discrimination due to race and sex faced by black women compared to black men and white women in the United States (U.S.)^22^. Stress exposure due to e.g. racism or sexism and subsequent coping mechanisms can result in higher morbidity and mortality^23^. Besides experiences of discrimination on the individual level, multiple discrimination can occur on a structural level diminishing peoples’ social power and opportunity for political participation^24^. In addition, agency to resist against one’s social categories might moderate the effect of intersections on health outcomes^25^. Thereby, social mechanisms can occur either additive (following the double/triple jeopardy) or multiplicative (in accordance with the multiple jeopardy) or some combination. Additive effects represent the sum of privileges and/or disadvantages, whereas multiplicative effects imply that the effects of the social characteristics enhance each other^25–27^.

Research on intersectional inequalities in different health outcomes, mainly self-rated mental and/or physical health status, received increased attention in the last five to ten years, currently still primarily in the U.S.^28,29^. Thereby, research focuses on the intersections of gender, race/ethnicity (history of migration) and/or socio-economic status (indicated by income, education and/or occupation). Due to the diverse assessment of the intersectional categories as well as methodological aspects, comparability is limited and results are inconclusive^28,29^. For the European or German context, so far only few studies were conducted indicating intersectional inequalities according to female gender, low income, and history of migration^30–32^. Common to all studies and in accordance with theoretical assumptions^21,22^, multiple privilege is attributed to the intersection of males with high income born in the respective country, whereas multiple disadvantage is attributed to females with low income born outside the respective country. These assumptions also serve as the basis for the present study.

To shed further light on the social context of SSS and to identify the ones with highest SSS, an intersectional approach is needed. Accordingly, we investigated the following research questions: To which degree is the adult population living in Germany affected by SSS? Is SSS more pronounced in people of female gender, low income, and history of migration, indicating social inequalities in SSS? If so, do gender, income and history of migration interact, indicating intersectional inequalities in SSS? And relatedly, have the more disadvantaged intersections (including female gender, low income, and a history of migration) significantly higher SSS than the more privileged ones (including male gender, high income, and no history of migration)?

## Methods

### Study design and sample

Analyses are based on cross-sectional data of the adult population (age ≥ 18 years) living in Germany. Data was collected via a telephone survey (computer assisted telephone interviews) from March until May 2022 by a company specialized on market and social research. By applying a dual frame approach with random-digit-dialing, 70% registered and computer generated as well as 30% mobile phone numbers were included in the sample^33^. Within households, respondents were randomly chosen via the Kish selection-grid^34^. Sample size calculation was based on a vignette design applied in the study (for details, please see the published study protocol^35^). These vignettes were not used in the present analyses. Interviews were conducted in German language. Accordingly, people with insufficient German language skills were excluded. The sample consisted of N = 2413 participants. The response rate was 45%. Data was weighted following a standardized three steps approach^33,36^. First, the distribution of household sizes in the sample was weighted according to the official distribution in the population. Second, design weights were calculated to correct for differing probabilities of selection rooted in the sampling design, including the household size and number of household and mobile phone numbers. Third, continuing bias in the distribution of socio-demographic characteristics due to e.g. higher non-response of certain groups of people was adjusted by applying the iterative proportional fitting including age, gender, education, and place of residence. After the three-steps weighting approach, the weighted study sample, regarding the fitted variables age, gender, and education (see Table 1) corresponded to the socio-demographic distribution of the official 2022 German statistics. Informed consent was obtained from all subjects and/or their legal guardian(s).

**Table 1:** Socio-demographic characteristics of the study population in comparison with official German statistics (%).

	Weighted sample (n = 2411)	Official German statistics	p*
Gender		^a^	
Female	51.1	50.7	0.717
Male	48.9	49.3	
Age groups		^b^	
18–40	32.2	33.3	0.471
41–60	34.0	33.1	
≥ 61	33.9	33.6	
Level of education		^c^	
Low (≤ 9 years)	31.2	30.2	0.677
Medium (10 years)	31.3	31.7	
High (≥ 12 years)	37.7	38.1	
Equivalent income (in Euro)^1^			
Low (≤ 1250)	34.7	–	
Medium (1251– < 2250)	30.1	–	
High (≥ 2250)	35.2	–	
History of migration			
No history of migration	77.6	–	
People whose parent(s) immigrated	11.2	–	
People who immigrated themselves	11.3	–	

*Chi^2^-test. —Data not available for population aged 18 years or older.

^a^Federal Statistical Office 2023^50^.

^b^Federal Statistical Office 2023^51^.

^c^Federal Statistical Office 2023^52^.

The survey is part of a large interdisciplinary project on persistence of somatic symptoms. The framework and study design of the Research Unit 5211 “Persistent SOMAtic Symptoms ACROSS Diseases: From Risk Factors to Modification (SOMACROSS)” and the subproject “Social Inequalities in Aggravating Factors of Persistent Somatic Symptoms (SOMA.SOC)” have been described in detail in the published study protocols^35,37^.

### Measures

Social inequalities: Age, gender, education, net household income per month, and history of migration were assessed by a standardized questionnaire. Since only two respondents reported their gender as diverse, they were excluded from the analyses and the variable was dichotomized into male and female. The net monthly household income was converted into the equivalent income to take differences in household size and composition into account. First, to convert the categorical income variable into a metric scale, the range of each category was replaced by the respective mean value. In case of the lowest category, the mean value was set at 750 Euro and for the highest category at 5250 Euro. The values were then weighted according to the household size (first person factor 1, other persons aged ≥ 15 years factor 0.5, children aged < 15 years factor 0.3)^38^. The equivalent income was divided into terciles with the following categories: low (≤ 1250 Euro), medium (1251– < 2250 Euro), and high (≥ 2250 Euro) income. Years of education was categorized according to the International Standard Classification of Education into the following three levels of education: low (≤ 9 years), medium (10 years), and high (≥ 12 years)^39^. According to the definition of migration background by the Federal Statistical Office^40^, history of migration was assessed based on the nationality (German nationality and/or another) and country of birth of participant as well as the country of birth of both parents (born in Germany yes/no). Accordingly, participants could be allocated to the following three categories: no history of migration, people whose parents (one or both) have immigrated, and people who have immigrated themselves. Due to the historical context of so called guest workers, late emigrants, and refugees, German research mostly assesses history of migration instead of race/ethnicity^23,40^.

Somatic symptom severity (SSS): To assess SSS, the German version of the Somatic Symptom Scale-8 (SSS-8)^9^ was used as the short version of the Patient Health Questionnaire (PHQ-15) . The scale consists of eight items asking about the most frequent somatic symptoms, using a time interval of the last seven days. Each item can be scored on a 5-point Likert scale (0 = not bothered at all to 4 = bothered very much). The total score of the sum scale ranges from 0 to 32 with higher scores indicating higher SSS. The German version of the SSS-8 has good internal consistency with Cronbach α = 0.81^9^. In the present study, Cronbach α was 0.82. Additionally, to assess different somatic symptom patterns, the eight items of the SSS-8 can be assigned to four subscales: gastrointestinal (1 item: stomach or bowel problems), pain (3 items: back pain; pain in arm, legs, joints; headache), cardiopulmonary (2 items: chest pain or shortness of breath; dizziness), and fatigue (2 items: feeling tired or having low energy; trouble sleeping). For comparability, the mean of each of the three subscales containing two or three items (pain, cardiopulmonary, fatigue) was weighted according to the number of items. The score of the four subscales ranges from 0 to 4.

### Missing data

In total, about 18% of individual items across all variables were missing (at random). This was mostly due to missing values on the income variable. For the other variables, the amount of missing values was about 3%. The missing data pattern was analyzed, and missing data was imputed using the multivariate imputation by chained equations method^41^. The method for imputing missing values depends on the variable’s nature. For continuous variables, predictive mean matching was applied, while logistic regressions were used for binary variables.

### Statistical analyses

To provide an overview of the study sample in terms of SSS, we first calculated arithmetic means with standard deviations (SD) for the SSS-8 sum scale and the four subscales. We then tested the hypothesis of higher SSS (in higher age, female gender, low income, and history of migration with analyses of variance.

Second, to gain a deeper understanding of (intersectional) social inequalities in SSS, we conducted multivariate analyses. As intersectional approaches have a high degree of complexity, we further analyzed the sum scale of the SSS-8 and refrained from including the subscales. We initially conducted a multiple linear regression model including the SSS-8 sum scale as the dependent variable, gender, income, and history of migration as predictors, and age as a covariate. To explore intersectional social inequalities in SSS, we then applied a descriptive intercategorical approach with multiplicative scale interaction^24^. Therefore, a second linear regression model was conducted with the SSS-8 sum scale as the dependent variable, gender, income, and history of migration as a three-way interaction term, and age as a covariate. Thereby, the intersections of gender, income, and history of migration represent not only characteristics on individual level, but broader social contexts^24^. Checks for multicollinearity revealed no collinearity issues. Concerning homoscedasticity and multivariate normality, deviation of residuals was observed due to the right-skewed distribution of the outcome. Hence, robust standard errors were used to ensure heteroscedasticity-consistent estimation^42^. Estimated marginal means with robust standard errors were calculated based on the multiple linear regression model including intersections. To identify the intersection with highest SSS, pairwise comparison of the estimated marginal means between the 18 intersections of gender, income, and history of migration (18 = 2 × 3 × 3) was carried out. Thereby, males with high income and no history of migration were set as the intersection of highest privilege, whereas females with low income who immigrated themselves were hypothesized as the most disadvantaged intersection. All other intersections represented a mix of privilege and disadvantage. To consider multiple testing, p-values were corrected with the false discovery rate according to Benjamin & Hochberg^43^. Performance of the different models (including explained variance and the interclass correlation coefficient, among others) was compared and tested with Vuong’s test to see if including the three-way interaction term of gender, income, and history of migration in the linear regression model was beneficial^44,45^. To assess the type of underlying social mechanism, we refer to significant interaction terms and Vuong’s test^25^. For all analyses, the weighted data set was used. The significance level for p-values was set at p < 0.05. Analyses were conducted with the R packages “sjPlot”^46^, “ggeffects”^47^, “easystats”^48^, and “ggplot2”^49^.

## Ethical approval

The study design was approved by the Ethics Commission of the Hamburg Medical Chamber (No. 2020-10194-BO-ff). All research was performed in accordance with relevant guidelines/regulations and the Declaration of Helsinki.

## Results

As presented in Table 1, mean age of participants was 51.4 years (SD = 18.8). Gender was equally distributed. About one third of respondents had a low (≤ 9 years), medium (10 or 11 years) or high education (≥ 12 years). The distribution of age, gender, and education corresponds to official German statistics^50–52^. The equivalent income of about one third of respondents could be rated as low (≤ 1250 Euro), medium (1251– < 2250 Euro) or high (≥ 2250 Euro). About 20% of participants had a history of migration. For the latter two, comparison with official German statistics was not possible since data was not available for the population aged 18 years or older^53,54^.

Means of the SSS-8 sum scale and the four subscales are presented in Table 2. Mean of the SSS-8 sum scale was 5.8 (SD = 5.7). Regarding the four subscales, respondents were more bothered by fatigue (mean = 1.1, SD = 1.1) and pain-related symptoms (mean 0.8, SD = 0.9) compared to gastrointestinal (mean = 0.5, SD = 0.9) or cardiopulmonary symptoms (mean 0.4, SD = 0.7).

**Table 2:** Socio-demographic characteristics and somatic symptom severity (SSS-8; n = 2411; mean (standard deviation)).

	Sum scale^a^		Subscales^b^							
		p^*^	Gastro	p^*^	Pain	p^*^	Cardio	p^*^	Fatigue	p^*^
Overall	5.83 (5.70)		0.49 (0.91)		0.82 (0.86)		0.39 (0.73)		1.05 (1.08)	
Gender										
Male	5.28 (5.52)	** < 0.001**	0.43 (0.84)	**0.001**	0.74 (0.81)	** < 0.001**	0.38 (0.74)	0.551	0.92 (1.03)	** < 0.001**
Female	6.39 (5.83)		0.55 (0.98)		0.90 (0.89)		0.39 (0.72)		1.17 (1.11)	
Age groups										
18–40	5.28 (5.31)	**0.003**	0.50 (0.89)	0.712	0.67 (0.75)	** < 0.001**	0.31 (0.68)	** < 0.001**	1.07 (1.05)	**0.002**
41–60	6.24 (6.29)		0.50 (0.95)		0.89 (0.94)		0.40 (0.78)		1.13 (1.17)	
≥ 61	5.93 (5.42)		0.47 (0.89)		0.89 (0.85)		0.44 (0.72)		0.95 (1.01)	
Equivalent income (in Euro)										
High (≥ 2100)	4.74 (4.72)		0.37 (0.91)		0.63 (0.71)		0.34 (0.67)		0.90 (0.99)	** < 0.001**
Medium (1251– < 2100)	5.14 (4.88)		0.42 (0.84)		0.76 (0.80)		0.30 (0.61)		0.93 (0.98)	
Low (≤ 1250)	7.27 (6.69)	** < 0.001**	0.65 (1.03)	** < 0.001**	1.03 (0.97)	** < 0.001**	0.49 (0.84)	** < 0.001**	1.27 (0.99)	
History of migration										
No history of migration	5.50 (5.43)	** < 0.001**	0.45 (0.89)	** < 0.001**	0.77 (0.82)	** < 0.001**	0.36 (0.69)	**0.006**	1.01 (1.06)	** < 0.001**
People whose parent(s) immigrated	7.08 (5.92)		0.58 (0.95)		1.02 (0.93)		0.47 (0.80)		1.26 (1.10)	
People who immigrated themselves	6.82 (6.95)		0.66 (1.02)		0.96 (1.00)		0.48 (0.87)		1.16 (1.18)	

^a^Range 0–32. ^b^Range 0–4. ^*^Analysis of variance.

Analyses of variance revealed significant differences in SSS within socio-demographic variables. Highest scores were found in females, middle aged people (41–60 years), people with low income, and people whose parent(s) immigrated. Similar patterns were found for the four subscales; Except no significant (p = 0.712) age difference showed for the gastrointestinal subscale and no significant (p = 0.551) gender difference appeared for the cardiopulmonary subscale. Moreover, for both of these subscales, highest values were found in people who immigrated themselves. Altogether, these bivariate analyses revealed social inequalities in SSS.

These social inequalities in SSS were also found in the multiple linear regression model 1 (Table 3). Age, female gender, low income, and history of migration were all significant predictors of higher SSS. The explained variance was 6.0%. Table 3 additionally presents model 2 including the interaction terms of gender, income, and history of migration. Here, female gender, low income and people who immigrated themselves remained significant predictors of higher SSS. Additionally, these predictors were significant in certain interaction terms, namely female*low income (p = 0.035), low income*parent(s) immigrated (p = 0.002), and female*low income*parent(s) immigrated (p < 0.001). The explained variance was 7.2%. Moreover, model 2 showed better performance than model 1 although Vuong’s test did not reach statistical significance (p = 0.052; see Supplementary Table S2).

**Table 3:** (Intersectional) social inequalities in somatic symptom severity (SSS-8): multiple linear regression model 1 including gender, income, and history of migration as predictors and model 2 additionally including the interaction terms of gender, income, and history of migration (n = 2411).

	Model 1			Model 2		
	B	Beta	p	B	Beta	p
(Intercept)	3.21	NA	< 0.001	3.19	NA	< 0.001
Age	0.02	0.07	**0.019**	0.02	0.07	**0.001**
Female gender^a^	0.99	0.09	**0.001**	1.08	0.09	**0.001**
Income medium^b^	0.07	0.01	0.337	0.65	0.05	0.145
Income low	2.17	0.18	** < 0.001**	1.21	0.10	**0.008**
People whose parent(s) immigrated^c^	1.61	0.09	**0.001**	0.57	0.03	0.562
People who immigrated themselves^c^	1.24	0.07	**0.002**	1.75	0.10	**0.043**
Female*income medium				− 0.98	− 0.06	0.116
Female*income low				1.32	0.09	**0.035**
Female*parent(s) immigrated				1.81	0.07	0.225
Female*immigrated themselves				− 1.54	− 0.06	0.238
Income medium*parent(s) immigrated				0.40	0.01	0.758
Income medium*immigrated themselves				− 0.81	− 0.02	0.522
Income low*parent(s) immigrated				3.82	0.14	**0.002**
Income low*immigrated themselves				− 0.15	− 0.01	0.901
Female*income medium*parent(s) immigrated				− 1.42	− 0.03	0.468
Female*income medium*immigrated themselves				0.95	0.02	0.638
Female*income low*parent(s) immigrated				− 6.63	− 0.17	** < 0.001**
Female*income low*immigrated themselves				1.58	0.05	0.353
R^2^			0.060			0.072

NA not applicable.

^a^reference: male. ^b^reference: high income. ^c^reference: no history of migration.

Table 4 presents the age-adjusted estimated marginal means (emm) with 95% confidence intervals (CI) of SSS in the 18 intersections of gender, income, and history of migration based on the multiple linear regression model 2. The three intersections with highest SSS were males with low income whose parent(s) immigrated (emm = 9.83, 95%CI 6.58–13.07), females with low income who immigrated themselves (emm = 9.47, 95%CI 6.88–12.05), and females with low income and no history of migration (emm = 7.83, 95%CI 6.70–8.96). In contrast, males with high income and no history of migration (emm = 4.22, 95%CI 3.66–4.78) had lowest SSS, followed by males with high income whose parent(s) immigrated (emm = 4.40, 95%CI 3.48–6.12), and males with medium income and no history of migration (emm = 4.87, 95%CI 4.04–5.71). Significant differences between the above mentioned intersections with highest and lowest SSS became also apparent in the post-hoc pairwise comparison of the estimated marginal means of all 18 intersections with false discovery rate controlling (see Supplementary Table S1, with +/− indicating significant differences between the intersections with highest and lowest SSS).

**Table 4:** Estimated marginal means (emm) of somatic symptom severity (SSS-8) in the intersections of gender, income, and history of migration (n = 2411; adjusted for age).

Intersection	Estimated marginal mean	95% confidence interval	Proportion of intersections in the sample(%)
MnMhI	4.22	3.66–4.78	16.52
M2ndMhI	4.80	3.48–6.12	1.40
MnMmI	4.87	4.04–5.71	10.41
FnMmI	4.97	4.30–5.64	13.34
FnMhI	5.30	4.61–5.99	12.79
F1stMmI	5.31	3.78–6.84	0.92
MnMlI	5.44	4.41–6.46	9.69
F1stMhI	5.51	3.50–7.51	1.45
M1stMmI	5.81	3.56–8.07	1.70
M2ndMmI	5.84	4.18–7.50	2.23
M1stMhI	5.97	4.18–7.77	1.89
F2ndMmI	6.32	4.41–8.23	1.51
M1stMlI	7.04	2.28–11.80	2.32
F2ndMlI	7.39	5.46–9.32	2.15
F2ndMhI	7.68	4.99–10.37	1.10
FnMlI	7.83	6.70–8.96	14.84
F1stMlI	9.47	6.88–12.05	2.98
M2ndMlI	9.83	6.58–13.07	2.77

M male, F female, nM no history of migration, 2ndM people whose parent(s) immigrated, 1stM people who immigrated themselves, hI high income, mI medium income, lI low income.

## Discussion

### Summary of main results and discussion of current state of research

With our study, we shed further light on the social context of SSS in Germany. By applying an intersectional approach, we combined three important social determinants of health, namely gender, income, and history of migration, which were previously examined separately, if at all. Thereby, differentiating between parent(s) immigration and own immigration provides new insights into the intersections of people with a history of migration which were mostly divided into born inside/outside of the respective country in previous intersectional research^28,29^.

Our analyses revealed intersectional social inequalities in SSS, indicated by the significant interaction terms. The significant three-way interaction term female*low income*parent(s) immigrated reflects unexpected low SSS in this intersection. Intersections with highest SSS were males with low income whose parent(s) immigrated, females with low income who immigrated themselves, and females with low income and no history of migration. The intersections with lowest SSS were males with high income and no history of migration, males with high income whose parent(s) immigrated, and males with medium income and no history of migration. In post-hoc pairwise comparison, the intersections with highest and lowest SSS differed significantly from each other.

The score of the most affected intersection (emm = 9.83) was more than twice as high compared to the least affected intersection (emm = 4.22). According to the validation study by Gierk et al.^9^, this corresponds to an increase of the SSS-8 severity category from low (4–7 points) to medium (8–11 points). This is relevant for health care, since in the validation study, an increase of each SSS-8 severity category was associated with a 53% (95%CI 44–63%) increase in health care visits. In addition, the difference of the highest and lowest emm (9.83–4.22 = 5.61) can be rated as clinically relevant, since the minimal clinically important difference of the SSS-8 was estimated as a 3-point change^12^. As hypothesized, the intersection a priori defined as the most privileged one, namely males with high income and no history of migration, had lowest SSS. However, the intersection a priori set as the most disadvantaged, namely females with low income who immigrated themselves, showed (only) second highest SSS. In the intersections of mixed privilege and disadvantage no clear trend emerged. For instance, males whose parent(s) immigrated with low income had highest SSS but second lowest for the ones with high income. In relation to the impact of the three social characteristics, all intersections including low income (except males with no history of migration) were found among the ones with highest SSS. In contrast, no such trend emerged regarding gender or history of migration. Therefore, our study revealed further evidence for the great importance of economic deprivation in the light of other social characteristics like history of migration^23^.

Regarding the type of social mechanism (additive and/or multiplicative effect), inconclusive patterns emerged. On the one hand, there was indication for a multiplicative effect, since the multiple linear regression model included some significant interaction terms and showed better model performance^25^. On the other hand, the (just about) insignificant Vuong’s test confirmed no benefit of the three-way interaction term in the linear regression model. However, as currently discussed, the latter does not disprove intersectionality in SSS, “as it is above all a framework to understand heterogeneity and social power rather than a hypothesis”^25^.

For the European or German context, only few intersectional analyses were conducted so far indicating different intersectional gradients in several health outcomes. Since these studies differentiate between people born in and outside of the respective country (instead of race/ethnicity in case of studies from the U.S.), they are particularly suitable to contextualize our findings regarding history of migration. For instance, Wandschneider et al.^30^ found an intersectional gradient of female sex, feminine gendered practice and immigration experience associated with worse subjective mental and physical health in Germany. For Sweden, Wamala et al.^32^ found an intersectional gradient of female gender, low income, and birth outside of Sweden in acute somatic symptoms. However, contrary to our findings, the study revealed a clear intersectional gradient and the highest burden was found among women with high income who were born outside Sweden. In another Swedish study by Wemrell et al.^31^, similar to our findings, the worst self-rated health was found in females with low income who were born outside of Sweden. More intersectional research is needed to provide further insights into the diverse social (intersectional) contexts regarding health outcomes.

The intersectional inequalities in SSS found in our study can be explained in two directions. First, as somatic symptoms are reported in various diseases, an unequal distribution of these underlying diseases may contribute to an unequal distribution of SSS. For instance, individuals with lower income have a higher burden of chronic diseases^55^, females with no history of migration are more affected by chronic physical diseases like coronary heart disease, and females with a history of migration more often suffer from mental disorders like depression^56^. All these diseases are related to higher SSS^3,4,9^. Furthermore, an association between a longer duration of stay in the country of immigration and a worse subjective health was found^23^. Nevertheless, it is important to acknowledge that not only the history of migration itself but associated socio-economic disadvantages and experiences of discrimination are responsible for higher health burden, as discussed above^23^. Psychosocial factors like lack of social support, traumatic or adverse life events, and loneliness revealed to be strong predictors of somatic symptoms^57^. Since these psychosocial factors in turn are closely linked to social disadvantage and immigration experiences, they might also contribute to higher SSS in more disadvantaged intersections^20,23^.

### Limitations

Some methodical aspects have to be considered when interpreting our results. The study sample with N = 2413 made discriminating intersectional analyses possible. Nevertheless, in the 18 intersections, there was quite some variation in the number of respondents (see Table 4). For this reason, we refrained from stratifying our analysis by age but presented age-adjusted results. Since age was associated with SSS in the bivariate analyses as well as the multiple linear regression models, it can be assumed that there are important differences according to individual life stages, which we were not able to take into account. Future studies with larger sample sizes could solve this problem. Also, the participants who reported their gender as diverse had to be excluded from analyses due to insufficient sample size, although additional differentiations of gender would have been preferable^21^. In future studies, gender practice and/or sexual orientation could also be analyzed.

Furthermore, our study sample did not fully cover the population with a history of migration^53^, especially with own migration experiences, last but not least because insufficient German language skills were an exclusion criterion in the present study. Since history of migration showed to be a significant predictor of SSS, associations might be even stronger for some subgroups like refugees. Future migration-related research should cover different languages and focus on lived experiences of specific migration populations to address heterogeneity^23^. In 2023, the focus on migration-related experiences rather than the country of birth was included in the definition of the Federal Statistical Office based on recommendations of the Commission on Integration^58^. In light of the research aim of the present study a pairwise comparison based on a fixed effects model with interaction terms was reasonable to compare the social groups with highest and lowest SSS^24,25^. However, to further address heterogeneity and gain a more comprehensive understanding on underlying social mechanisms, random effects models like MAIHDA (multilevel analysis of individual heterogeneity and discriminatory accuracy) seem promising in quantitative health research to identify differences between and within intersections^25,59^. In adition, to explore the richness of individual experiences within intersections qualitative methods appear promising^25^.

Moreover, it can be assumed that crucial protective factors (like agency, resilience, social support), risk factors (like experiences of individual or structural discrimination) as well as individual chronic illness would have drawn a more comprehensive picture of the predictors of SSS. This is also reflected in the low explained variance. However, these additional factors were not focus of the present study but could be included in future studies as discussed above. Furthermore, to reduce collinearity, we solely chose income (and not education in addition) as an indicator of socio-economic status as income not only effects material conditions but also social participation^60^.

With a mean SSS-8 score of 5.8 (SD = 5.7), we found slightly higher SSS in our study population compared to the initial validation study^9^. This may be explained by a slightly higher mean age and a lower income level of our study population. Additionally, since the study is specific for the German context, transferability of results to other countries is limited.

## Conclusion

Intersectionality examines the interaction of multiple forms of disadvantage or oppression and the influence on peoples’ (health) experiences. By applying this framework, the study revealed intersectional inequalities in SSS as well as the intersections with highest SSS, namely males with low income whose parent(s) immigrated, females with low income who immigrated themselves, and females with low income with no history of migration. Following the proportionate universalism, a combination of universal and target interventions located in the health care system seems promising to reduce health disparities related to SSS (besides the general effort to reduce income inequalities). These may consist of brief universal screening tools for SSS in different languages to identify the individuals affected by high SSS in combination with targeted treatment interventions. Future intersectional research should emphasize the lived experiences of disadvantage and privilege and apply random effects models as well as qualitative approaches to shed further light on the social context of SSS.

### Supplementary Information

Supplementary Information.

## Data Availability

The datasets generated in and/or analyzed in the current study are available from the corresponding author on reasonable request.
