# Supplementary material for: Intersectional inequalities in somatic symptom severity in the adult population in Germany found within the SOMA.SOC study
Source: Sci Rep. 2024 Feb 15;14:3820. doi: 10.1038/s41598-024-54042-8 (PMC10869707; doi:10.1038/s41598-024-54042-8)
Supplement: Supplementary file 1 — Supplementary Information. [file 41598_2024_54042_MOESM1_ESM.pdf]

Intersectional inequalities in somatic symptom severity in the adult population in Germany found within the SOMA.SOC study

Rieke Barbek, Anne Toussaint, Bernd Löwe, Olaf von dem Knesebeck

Supplementary Information

Table S1: Pairwise comparison of estimated marginal means of somatic symptoms severity (SSS-8) in the intersections of gender, income, and history of migration with false discovery rate-controlling and robust standard deviations (n =2,411).

	MnMhI	MnMmI	MnMlI	M2ndMhI	M2ndMmI	M2ndMlI	M1stMhI	M1stMmI	M1stMlI	FnMhI	FnMmI	FnMlI	F2ndMhI	F2ndMmI	F2ndMlI	F1stMhI	F1stMmI	F1stMlI
MnMhI<br>male*<br>no history of migration*<br>high income						-						-			-			-
MnMmI<br>male*<br>no history of migration*<br>medium income						-						-						-
MnMlI<br>male*<br>no history of migration*<br>low income												-						-
M2ndMhI<br>male*<br>parent(s) immigrated*<br>high income						-						-						-
M2ndMmI<br>male*<br>parent(s) immigrated*<br>medium income																		
M2ndMlI<br>male*<br>parent(s) immigrated*<br>low income	+	+		+							+							
M1stMhI<br>male*<br>immigrated themselves*<br>high income																		
M1stMmI<br>male*<br>immigrated themselves*<br>medium income																		
M1stMlI<br>male*<br>immigrated themselves*<br>low income																		

	MnMhI	MnMmI	MnMlI	M2ndMhI	M2ndMmI	M2ndMlI	M1stMhI	M1stMmI	M1stMlI	FnMhI	FnMmI	FnMlI	F2ndMhI	F2ndMmI	F2ndMlI	F1stMhI	F1stMmI	F1stMlI
FnMhI female* no history of migration* high income												-						-
FnMmI female* no history of migration* medium income						-						-						-
FnMlI female* no history of migration* low income	+	+	+	+						+	+							
F2ndMhI female* parent(s) immigrated* high income																		
F2ndMmI female* parent(s) immigrated* medium income																		
F2ndMlI female* parent(s) immigrated* low income	+																	
F1stMhI female* immigrated themselves* high income																		
F1stMmI female* immigrated themselves* medium income																		
F1stMlI female* immigrated themselves* low income	+	+	+	+						+	+							

Adjusted for age.

M male, F female, nM no history of migration, 2ndM people whose parent(s) immigrated, 1stM people who immigrated themselves, hI high income, mI medium income, lI low income.

+ significantly more SSS. - significantly less SSS. Empty fields no significant difference. Significance level $p < 0.05$ with false discovery rate correction.

Table S2: Comparison of model indices.

Name	R^2	R^2 adjusted	RMSE	Sigma	AIC weights[1]	AICc weights[1]	BIC weights[1]	Performance Score (%)
m1_initial	0.060	0.058	5.206	5.534	0.017	0.019	1.000	14.3
m2_intersections	0.072	0.065	5.196	5.511	0.983	0.981	0.000	85.7

Models: m_initial (lm) multiple linear regression model with gender, income, history of migration as predictors, and age as covariate. m_intersections (lm) multiple linear regression model with intersections of gender, income, and history of migration, adjusted for age.

Model indices: R^2 explained variance of dependent variable. R^2 adjusted for different number of predictors. RMSE Root mean square error as the average distance between predicted and actual values. Sigma standard error of residuals. AIC Akaike-Information-Criterion based on log-likelihood. AICc Interclass correlation coefficient. BIC Bayesian-Information-Criterion based on log-likelihood with sample size adjustment. Performance score Index composed of the mean values of all indices (rescaled from 0 to 1).

[1] wt = exp(-0.5 * delta_ic) / sum(exp(-0.5 * delta_ic)) with delta_ic = model index – smallest model index in the model set.

Higher values (except for RMSE) indicating better model fit to identify the model that best explains the dependent variable.

Non-significant Vuong's test (m_intersections vs m_initial p = 0.052).
